# The “Slash Pack” as a Temporary Method for Initial Hemorrhage Control of Penetrating Neck Trauma in the Emergency Department: A Report of Two Cases

**DOI:** 10.7759/cureus.85041

**Published:** 2025-05-29

**Authors:** Steven J Laxton, Jordan Yutzy, Tanner Whiting, Joe Pflederer, Jacqueline Dovgalyuk, Nina Fredericks, Eric Bruno

**Affiliations:** 1 Department of Emergency Medicine, University of Tennessee Health Science Center (UTHSC) - Nashville/Ascension Saint Thomas Rutherford, Murfreesboro, USA; 2 Department of Obstetrics and Gynaecology, University of Tennessee Health Science Center (UTHSC) - Nashville/Ascension Saint Thomas Rutherford, Murfreesboro, USA

**Keywords:** acute arterial hemorrhage, head and neck trauma, hemorrhagic shock, neck bleeding, neck laceration, neck trauma, penetrating neck trauam, penetrating neck trauma, penetrating trauma

## Abstract

Penetrating trauma to the neck poses a critical challenge in the emergency department. These wounds have the potential to lead to rapid blood loss, hemorrhagic shock, and an increase in mortality. Large academic tertiary centers typically have vast resources and consultants to allow for rapid assessment and treatment of penetrating neck trauma. In community emergency departments, this is not typically the case, as surgical backup is usually not readily available and lacks the additional subspecialty coverage to aid in the management of the trauma patient. This situation often necessitates innovative methods of achieving hemostasis in certain life-threatening situations. This report explores a novel approach, termed the “slash pack,” as a potential solution for managing such traumatic injuries. The report discusses two cases in which penetrating neck trauma was successfully managed with the slash pack technique in a community hospital for stabilization prior to the transfer of the patients to the regional tertiary trauma center. Both patients were stabilized and transferred to a level 1 trauma center for definitive care with complete resolution and no further sequelae from injury.

## Introduction

Penetrating trauma to the neck is a time-sensitive concern in emergency medicine that can result in rapid hemorrhagic shock and high mortality rates. This type of injury is caused by various mechanisms, including violence (such as stabbings and gunshot wounds) and accidents (such as motor vehicle collisions and work site accidents). Penetrating neck injuries account for approximately 5-10% of all trauma cases and carry an elevated mortality rate of up to 10% [1,2].

The neck is divided into three zones when performing a trauma assessment. Zone I refers to the lower part of the neck, which extends from the clavicles to the cricoid cartilage. This zone contains many vital structures, including the trachea, esophagus, and major blood vessels. Zone II is the middle portion of the neck from the cricoid cartilage to the angle of the mandible. This zone contains the important structures of the carotid artery, jugular vein, and several nerves. Zone III is the upper part of the neck extending from the angle of the mandible to the base of the skull. While each zone of the neck contains vital organs, with regards to trauma, typically zones I and II require immediate surgical intervention, while zone III often is able to be managed more conservatively or with angiography. The most commonly injured area of the neck is zone II, which can easily be accessed surgically. However, in zones I and III injuries, exposure and vascular control are more difficult to achieve [3]. The physical examination of patients with suspected neck trauma is crucial, as it can reveal life-threatening vascular injuries, potential esophageal or tracheal damage, which can lead to airway compromise.

Current diagnostic approaches for penetrating neck injuries include imaging studies, such as contrast-mediated CT angiography, to evaluate the extent of injury. However, sometimes the extent and severity of the trauma require immediate interventions to stabilize the patient. Standard management protocols emphasize direct pressure, packing, and topical hemostatic agents to control hemorrhage [4,5]. In community emergency departments that lack readily available surgical support, challenges arise in how a patient can be stabilized, making it paramount for healthcare providers in these situations to adopt innovative temporary measures to stabilize patients for transfer for definitive treatment with trauma surgery.

This case report is significant in that it introduces the “slash pack” technique as a novel technique of achieving hemostatic control in penetrating neck injuries. No prior report of penetrating neck injuries with arterial hemorrhage managed using this technique could be found in the literature. By utilizing this method, emergency departments can effectively manage severe hemorrhage when conventional methods are inadequate. These cases indicate the importance of having adaptable and effective strategies for emergency care, especially in settings without immediate access to trauma or vascular surgery, ultimately enhancing patient outcomes during critical moments of care.

## Case presentation

Case 1: laceration resulting from assault with a beer bottle

A 22-year-old male patient with no past medical history presented to the emergency department with a large laceration that extended from the posterior right ear to the inferior and lateral part of the neck following a physical altercation. The patient, who had consumed several alcoholic beverages prior to the incident, reported being struck in the neck by a broken beer bottle, leading to a large laceration to his right posterior neck that measured just over 10 cm in length with extensive subcutaneous tissue and muscle visualized. Emergency medical services (EMS) reported a large volume of blood loss on scene prior to being transported to the emergency department. On presentation, the patient was alert but significantly pale, with his clothes covered extensively in blood. He endorsed dizziness and was actively bleeding from a neck wound. The patient had a patent airway and was breathing and phonating appropriately.

Initial vital signs showed a heart rate of 93 beats per minute, blood pressure of 121/57 mmHg, respiratory rate of 24 breaths per minute, and oxygen saturation at 100% on room air. He was tachypneic, with a respiration rate of 24.

Initial examination showed a large, jagged laceration extending from the inferior and posterior to the right ear to the mid-posterior neck. The wound penetrated deep with extensive subcutaneous tissue and muscles visualized. There was also active arterial bleeding from two exposed vessels within the wound. The anterior neck was unremarkable, and neurological examination revealed no focal deficits. A rapid assessment of the wound led to immediate intervention for the traumatic hemorrhage. Hemostasis was initially achieved with direct pressure, followed by wound packing with a saline-soaked sterile roll of gauze. The gauze was then secured in the wound and tightly adhered to the wound with 2-0 nylon sutures using a running technique and four staples. The patient tolerated the procedure without complications, and hemostasis was achieved.

Initial laboratory results, including a complete blood count and basic metabolic panel, revealed no significant abnormalities to report. Due to significant acute blood loss, the patient was transfused with two units of emergency release O-negative packed red blood cells and a 1 L crystalloid fluid bolus. The patient was also given cefazolin for infection prophylaxis and a Tdap vaccine booster. Given the severity of the injury and the need for advanced trauma care, the patient was transferred to the regional level 1 trauma center for further management. He subsequently underwent surgical vascular intervention and achieved a full recovery without further sequelae.

After a fluid bolus and two units of blood transfusion, the patient maintained stable vital signs with improvement in tachypnea prior to transfer to a trauma center. 

Case 2: multiple stab wounds with cardiac arrest

A 28-year-old female patient with a medical history of hypothyroidism, chronic kidney disease stage II, and depression was brought to the emergency department via EMS following a stabbing. The patient was initially altered, combative, and hemorrhaging on scene and subsequently sedated, paralyzed, and intubated by EMS. The history of the incident was limited due to her clinical state and was provided by EMS. Initial examination revealed two large lacerations to the head and neck. The first noted was a large 12 cm linear laceration to the right scalp extending laterally across the forehead to the right lateral eye. Additionally, and most concerning, there was a 10 cm deep laceration present on the right posterolateral neck with active arterial hemorrhage and extensive exposure of subcutaneous tissues and muscles of the neck. 

The patient experienced a pulseless electrical activity (PEA) arrest shortly after arrival at the emergency department. Return of spontaneous circulation was achieved following three minutes of chest compressions and volume infusion. Immediate access was obtained via a right femoral large-bore venous catheter and a left femoral triple-lumen central catheter. The patient received fluid resuscitation with two units of O-negative blood and a 1 L crystalloid fluid bolus. Upon achieving return of spontaneous circulation (ROSC), bleeding resumed from the scalp and neck lacerations. The right scalp laceration was closed with staples. The more extensive and larger right neck wound was packed with a roll of sterile saline-soaked gauze. The gauze was then secured with staples and sutured in place. After resuscitation, the patient’s heart rate was between 90-100 beats per minute, and mean arterial pressure was between 60-65. She was initially hypothermic with a core temperature of 94°F and was treated with an active external rewarming device.

Imaging, including chest and pelvis X-rays, revealed no acute cardiopulmonary abnormalities or injuries to the pelvis. A ballistic projectile was noted at the level of the L3 vertebral body. A full survey was performed, and no gunshot wound was immediately obvious. An extended focused assessment with sonography for trauma (eFAST) exam revealed no free fluid in the abdomen or chest, no pericardial effusion, and no pneumothorax. The patient was administered a Tdap vaccine booster and was placed in a cervical collar for spinal immobilization. Due to the critical nature of the patient's condition, she was transferred to the regional level 1 trauma center for further evaluation via air ambulance. The patient was surgically managed, leading to recovery except right eye blindness requiring a right ocular prosthesis secondary to the scalp laceration that extended into and violated the right globe.

## Discussion

Penetrating neck injuries pose a significant challenge in the emergency department due to the dense concentration of vital structures, including the carotid arteries, jugular veins, trachea, and esophagus. Immediate and effective management is critical to reducing morbidity and mortality, particularly in cases involving major vascular injury and hemorrhage. Traditional management of penetrating neck injuries involves rapid hemorrhage control, airway protection, and surgical intervention when indicated [6]. This report examines the practicality and clinical uses of the slash pack as a novel technique for temporary and stabilizing hemostatic control in cases of significant vascular injury to the neck.

Penetrating neck injuries should be managed systematically according to Advanced Trauma Life Support (ATLS) protocols. A rapid primary and secondary survey is essential for the initial assessment of trauma. Early airway protection is also a critical early consideration for injury to the neck because of the elevated risk of airway compromise in this anatomical region [7]. Respiratory assessment should be performed promptly to evaluate for injuries such as pneumothorax and hemothorax. Hemodynamic stability must be rapidly assessed through monitoring of vital signs and peripheral pulses. Rapid identification and control of hemorrhage are crucial to reducing mortality.

Management of hemorrhage in penetrating neck injuries

One of the primary goals in managing penetrating neck injuries is to control hemorrhage, as it is a leading cause of death in these patients [8]. Immediate measures to achieve hemostasis include direct pressure, packing, and, when indicated, surgical exploration. In both cases, initial hemorrhage control was achieved through the use of the slash pack technique, which is different from packing as packing is used on small wounds that are amenable to being packed to achieve hemostasis whereas in this wound, which was quite large, it was not amenable to being able to simply pack it. 

The following are commonly described techniques that are used to control hemorrhage.

Direct Pressure

The first step in managing penetrating neck injuries is to apply direct pressure to the wound to control active bleeding. This may stabilize the patient temporarily while further definitive care is prepared [8].

Balloon Tamponade

Foley catheter balloon tamponade (FCBT) may be used, where the clinician inserts a Foley catheter into the wound and inflates it with 5 cc of water or until resistance is felt. Several studies have demonstrated that this intervention is highly successful in achieving hemostasis in hemorrhaging penetrating neck injuries [9,10]. 

Surgical Intervention

If bleeding persists despite interventions, surgical exploration is required to repair damaged vessels and other structures. Early surgical involvement is important in patients where hemostasis is not achieved [10].

Local Exploration

In the rare occasion where hemorrhage is not controlled using the above techniques and a surgeon is not immediately available, the emergency medicine physician may explore the wound and attempt to directly clamp the damaged vessel [11].

The slash pack technique

The "slash pack" is a novel technique that can be employed to achieve hemostatic control in penetrating neck injuries with active arterial hemorrhage. This method involves the application of saline-soaked rolled gauze directly over the bleeding site in large wounds, which is then secured in place with sutures or staples to tamponade hemorrhage. This technique combines the benefits of securing hemostatic material with the added advantage of localized compression. The slash pack is particularly advantageous in scenarios where immediate surgical intervention is not feasible, serving as an effective temporary measure to stabilize the patient until definitive surgical repair can be performed. It is especially useful in cases where patient transport is required, making the continued application of direct manual pressure challenging. 

**Figure 1:**
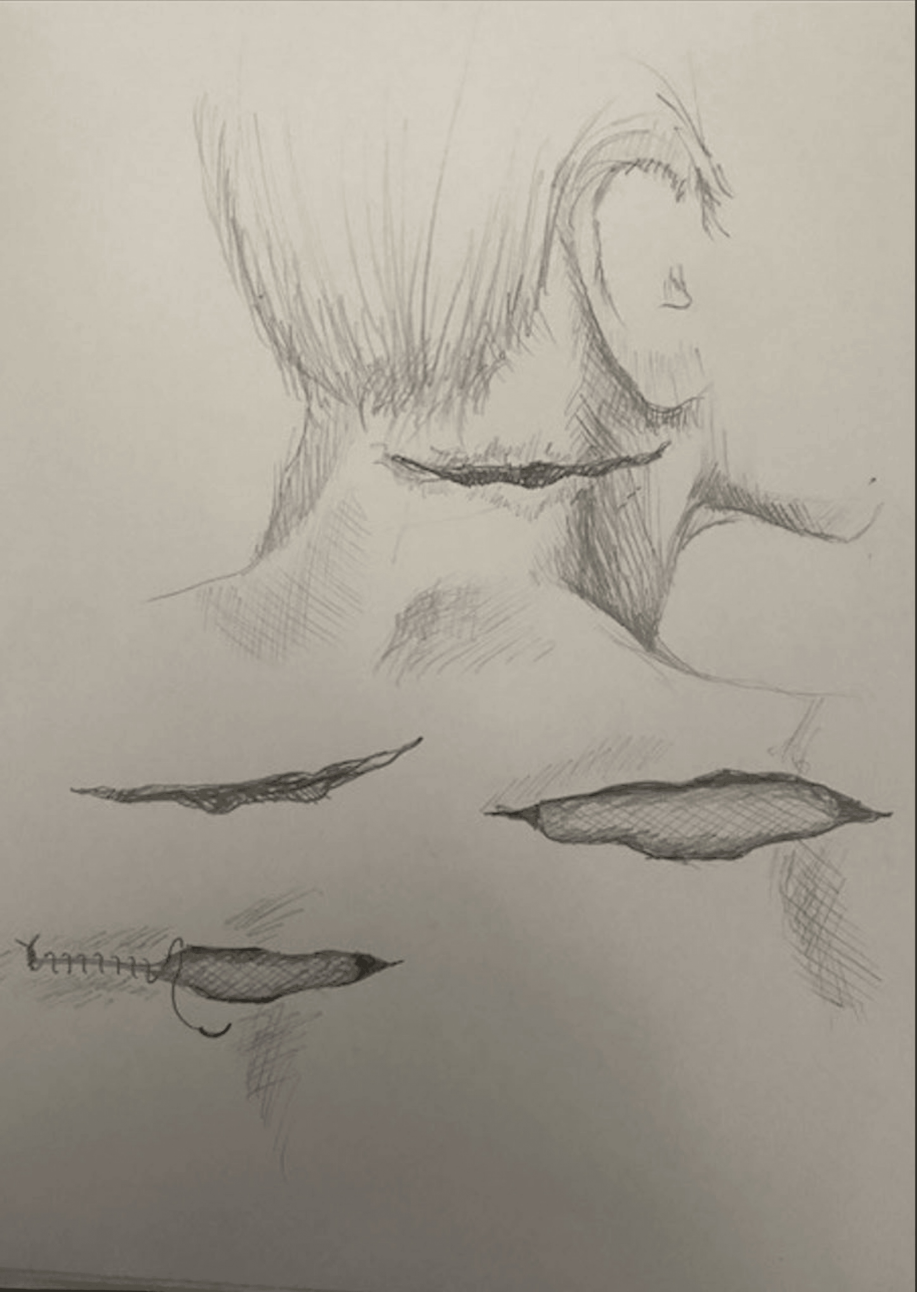
Medical illustration demonstrating the "slash pack" Original medical illustration demonstrating a large laceration to the posterolateral neck that was then packed with a roll of saline-soaked gauze with closure of the wound above it to tamponade hemorrhage. This illustration provides an example of the size and location of the laceration on the lateral-posterior neck that then had a roll of kerlix gauze inserted into the wound with closure of the wound over the top of the gauze. Illustration Credit: Nina Fredericks, MD

Both cases described in this report involved significant hemorrhage and hemodynamic instability, which were managed with the slash pack technique. Hemostasis was achieved in both patients, and both experienced favorable clinical outcomes at discharge. The slash pack technique proved to be a simple yet effective and potentially life-saving intervention for controlling hemorrhage in penetrating neck injuries while awaiting definitive surgical care. Figure 1 shows an illustration of this technique.

Anatomical zones of the neck

Identification of the anatomical zone involved in penetrating neck injuries significantly influences management and treatment strategies in the emergency department. While experienced clinicians may intuitively recognize the distinct risks associated with each cervical zone, a systematic and structured zone-based approach can sometimes minimize the risk of missed injuries and optimize patient outcomes [12].

**Figure 2:**
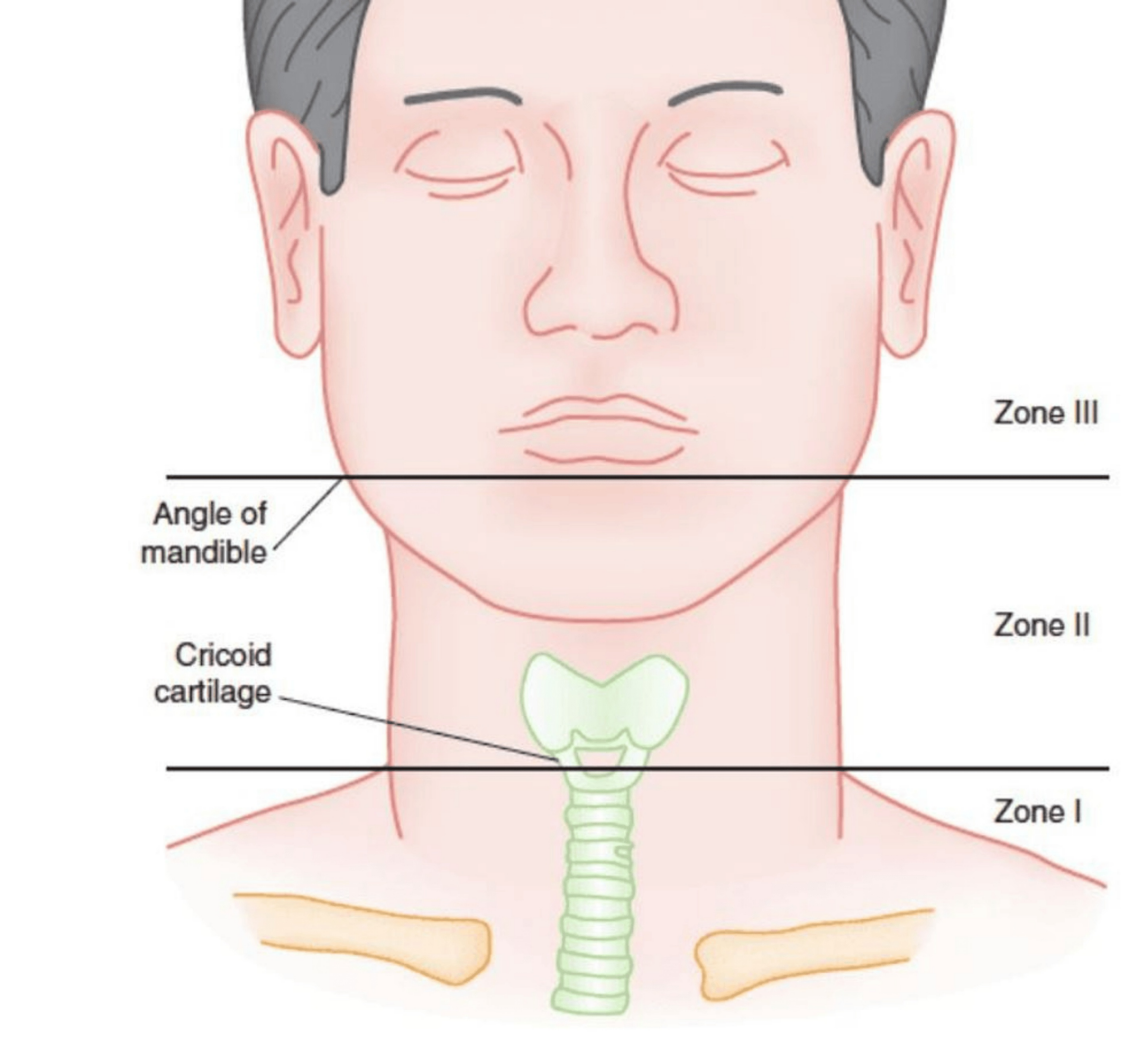
Anatomical zones of the neck showing the border of zones I, II, and III Image Source: RebelEM [13]; Distributed under CC BY-NC-ND 3.0, Creative Commons Attribution-NonCommercial-NoDerivs 3.0 Unported License. Permission for use was obtained prior to publication.

Zone I is the most caudal cervical zone, extending from the clavicles to the cricoid cartilage. It contains critical structures, including the subclavian arteries, lung apices, trachea, and esophagus. Zone II is the middle cervical zone, located between the cricoid cartilage and the angle of the mandible, encompassing the upper trachea, esophagus, larynx, and pharynx. Zone III is the most cranial zone, extending from the angle of the mandible to the base of the skull, and includes the salivary glands and multiple cranial nerves. The carotid and jugular vasculature traverse all three zones. Although zone II injuries are the most frequently encountered, zone I injuries carry the highest mortality due to the potential for mediastinal or intrapleural injury. Figure 2 shows an example of the commonly encountered anatomical zones of the neck with each border properly identified [13]. 

For many years, zone-based algorithms were employed to guide the management of penetrating neck injuries. However, new approaches have been adopted in recent years due to the frequent involvement of injuries spanning multiple zones, the presence of complex multi-zone injuries, and a high rate of negative surgical exploration. Advances in computed tomography imaging have facilitated more accurate identification of vascular and aerodigestive injuries, leading to increased adoption of a "no-zone" approach to management among surgeons. While anatomical zones have become less influential in the initial management within the emergency department, clinicians must remain cognizant of the three cervical zones and the distinct anatomical structures and injury risks associated with each [14].

Hard and soft signs

The presence of hard and soft signs remains a critical determinant in the management and disposition of patients with penetrating neck injuries. Hard signs, which indicate significant damage to major vascular, respiratory, or neural structures, necessitate immediate surgical exploration. These include active arterial bleeding, expanding hematoma, airway compromise, neurologic deficits, and shock unresponsive to resuscitation. Soft signs, such as minor hemoptysis, hoarseness, non-expanding hematoma, and stable subcutaneous emphysema, warrant a more conservative approach with close monitoring, further diagnostic evaluation, and consultation with surgical specialists [14,15]. Table 1 demonstrates applicable hard and soft signs that relate specifically to vascular injury.

**Table 1:** Physical exam findings that indicate hard signs of vascular injury specifically to the neck Table credit: Jordan Yutzy, MD

Hard signs of vascular injury
Severe or uncontrolled hemorrhage
Large, expanding, or pulsatile hematoma
Thrills or bruits
Shock unresponsive to fluid resuscitation
Absent or diminished radial pulse
Neurological deficit consistent with cerebral ischemia
Soft signs of vascular injury
Proximity wounds
Minor hemorrhage
Mild hypotension responsive to IV fluid resuscitation
Minor hemoptysis or hematemesis
Subcutaneous or mediastinal air
Non-pulsatile non-expanding hematoma
Dysphonia or dysphagia

Definitive management

Definitive management and treatment of penetrating neck injuries require the integration of the aforementioned principles, including zones of the neck, in addition to hard and soft signs. Hemodynamically unstable patients or those presenting with hard signs of vascular, aerodigestive, or neurological injury require immediate surgical exploration [16]. Conversely, patients exhibiting soft signs can often be managed more conservatively with close monitoring and early surgical consultation. The advent of computed tomography with angiography (CTA) has significantly improved diagnostic accuracy and management strategies. Studies evaluating the use of Foley catheter balloon tamponade for vascular injury have proposed several management algorithms, which may be extrapolated to the use of the slash pack for hemorrhage control [17]. These studies suggest that hemodynamically stable patients with successful initial hemorrhage control may safely undergo further investigation with CTA [18]. Early involvement of surgical services remains critical to optimize patient outcomes. Both patients in this report required transfer to a trauma center for further management due to the complexity of their injuries, highlighting the importance of timely and coordinated care in such cases.

Notes

It should be mentioned that this report is meant to provide techniques for community/non-trauma center emergency departments that can aid in stabilizing hemorrhage and maintaining the patient's native artery prior to evaluation by a trauma surgeon for definitive ligation or repair. We used saline-soaked gauze, and it is possible that dry gauze or gauze soaked in vasoactive drug (i.e., epinephrine) or tranexamic acid may be of benefit as well; however, there is no data available to know definitely which would be most effective. 

## Conclusions

Penetrating neck trauma has the potential to cause high morbidity and mortality in trauma patients. This can be due to critical vessel injury causing hemorrhagic shock, neurologic injury, or injury to other critical non-vascular structures such as the trachea. Current management includes application of pressure to resolve bleeding while further assessing based on physical exam findings and the zone location of the neck. CTA of the neck is the gold standard for evaluating vascular or structural injury in penetrating trauma to the neck. If the physical exam shows the presence of hard signs (vascular hemorrhage, expanding hematoma), then immediate surgical exploration is warranted, as this could lead to rapid decompensation. 

This report highlights the use of a novel and previously unreported technique that can be implemented in cases of penetrating trauma to the neck with vascular injury and arterial hemorrhage until the patient is able to obtain definitive treatment with vascular or trauma surgery. This report highlights the use of this technique to aid emergency clinicians and provide another tool in critical access areas or areas that require stabilization and transfer for definitive management. Areas that can be explored in further application of this method include the addition of epinephrine (or other vasoactive drug) or tranexamic acid-soaked gauze rolls, instead of saline-soaked gauze.
